# Oxytocin and the Default Mode Network: New Insights into Attachment and Self-Referential Processing: Oxytocin and the Default Mode Network

**DOI:** 10.31661/gmj.v14i.3812

**Published:** 2025-08-05

**Authors:** Shingo Ueda

**Affiliations:** ^1^ UNI H&H Graduate School Osaka 564-0051, Japan

**Keywords:** Oxytocin, Default Mode Network, Self-Referential Processing, Attachment, Social Cognition, Neuroimaging, Functional Connectivity, Neuropsychiatric Disorders

## Abstract

Recent advances in neuroscience have revealed a significant interplay between the neuropeptide oxytocin and the brain’s Default Mode Network (DMN), suggesting a pivotal role in modulating attachment and self-referential processing. This review synthesizes current findings from neuroimaging, behavioral studies, and clinical research to explore the “oxytocin-DMN axis” and its impact on social cognition and emotional regulation. We discuss how oxytocin influences intrinsic connectivity within the DMN, enhancing self-referential thought and social bonding by modulating key network nodes involved in autobiographical memory, empathy, and social awareness. Emerging evidence indicates that dysregulation in this axis may contribute to the neurobiological underpinnings of psychiatric disorders such as autism, social anxiety, and depression. Moreover, we evaluate the therapeutic potential of targeting oxytocin signaling pathways to restore or enhance DMN functionality. By integrating multidisciplinary perspectives, this review provides novel insights into the mechanistic links between hormonal modulation and intrinsic brain network dynamics, underscoring the importance of the oxytocin-DMN axis in the neurobiology of attachment and self-referential processing.

## Introduction

Social bonding and attachment represent fundamental aspects of human behavior and are central to our understanding of social cognition [1]. Emerging evidence highlights those neuropeptides, particularly oxytocin, play a critical role in modulating these behaviors through their actions on diverse neural circuits [2]. Oxytocin, a peptide synthesized in the hypothalamus and released both peripherally and centrally, has been associated with trust, empathy, and affiliative bonding [3]. Its influence extends to various domains of social and emotional processing, suggesting a key role in the regulation of interpersonal interactions and attachment behaviors.

Parallel to these developments, the Default Mode Network (DMN) has garnered significant attention in cognitive neuroscience due to its involvement in self-referential thought and introspection [4]. Comprising regions such as the medial prefrontal cortex, posterior cingulate cortex, and angular gyrus, the DMN is predominantly active during rest and is implicated in processes ranging from autobiographical memory to envisioning the future [5]. Recent neuroimaging studies have begun to delineate how oxytocin may modulate the functional connectivity of the DMN, thereby influencing self-related and social cognitive processes [6]. Evidence suggests that oxytocin administration can lead to alterations in DMN connectivity, potentially underpinning its effects on social perception and emotional regulation [7][8].

Understanding the intricate interplay between oxytocin signaling and DMN activity is essential, particularly given its potential implications for psychiatric conditions characterized by deficits in social functioning and abnormal self-referential processing [9][10]. Disorders such as autism spectrum disorder, social anxiety, and depression have been linked to both dysregulated DMN connectivity and aberrant oxytocin signaling [11]. These converging lines of research are not only advancing our knowledge of the neurobiological substrates of social behavior but are also paving the way for the development of novel, targeted interventions [12].

This review aims to synthesize current evidence on the oxytocin-DMN axis, exploring its mechanistic role in the neurobiology of attachment and self-referential processing [13]. By integrating findings from neuroimaging, behavioral studies, and clinical research, we discuss emerging therapeutic targets and highlight the potential of oxytocin-based interventions to modulate dysfunctional neural networks in mental health disorders [14]. Ultimately, the goal is to enhance our understanding of neuroendocrine regulation within the brain and to inspire future research into novel treatment strategies that address the core deficits in social cognition [15].

### Oxytocin in Neurobiology and Social Behavior

Oxytocin is a neuropeptide synthesized primarily in the hypothalamic paraventricular and supraoptic nuclei and exerts widespread effects on both central and peripheral systems [16]. Its release into the brain occurs through a well-coordinated process involving dendritic and axonal secretion, enabling oxytocin to modulate various neural circuits that underlie social behavior [17]. The oxytocin receptor (OXTR), a G protein-coupled receptor, is widely distributed across key brain regions such as the amygdala, nucleus accumbens, and prefrontal cortex, which are critical for processing social cognition and emotional regulation [18]. Activation of these receptors initiates intracellular signaling cascades that influence synaptic plasticity and neural connectivity, thereby shaping circuits involved in affiliative behavior and bonding [19].

Animal studies have provided compelling evidence for oxytocin’s role in regulating social behaviors including maternal care, pair bonding, and social recognition [20]. Experimental manipulations of oxytocin signaling in rodents, whether via genetic or pharmacological methods, often result in profound alterations in social memory and affiliative interactions, underscoring the evolutionarily conserved function of oxytocin in social behavior [21]. Complementary research in non-human primates has shown that oxytocin administration can enhance prosocial behaviors, suggesting a potential translational relevance for understanding human social cognition [22].

In human research, neuroimaging studies have revealed that intranasal oxytocin administration can increase neural activity in regions associated with empathy, social reward, and emotional regulation [23]. These neurophysiological findings are consistent with behavioral studies demonstrating that oxytocin enhances trust and improves the recognition of emotional expressions in social contexts [24]. Moreover, genetic studies have implicated polymorphisms in the OXTR gene with individual differences in social cognitive abilities and susceptibility to psychiatric conditions marked by social deficits [25].

Further supporting its clinical relevance, dysregulation in oxytocin signaling has been linked to neuropsychiatric disorders such as schizophrenia, autism spectrum disorder, and social anxiety [21]. The modulatory effects of oxytocin on key brain regions offer promising avenues for therapeutic intervention. Ongoing research is focusing on optimizing oxytocin-based treatments by refining dosing strategies and identifying specific patient populations that may derive the greatest benefit from such interventions [22][23].

In addition to its established role in social behavior, oxytocin has been found to interact with other neurotransmitter systems, including dopamine and serotonin, which are also implicated in reward processing and mood regulation [16]. These interactions suggest that oxytocin’s influence may extend beyond social cognition to impact broader neural networks, potentially enhancing its therapeutic efficacy in treating complex neuropsychiatric disorders [24][25].

Overall, the neurobiological foundations of oxytocin’s role in social behavior highlight its potential as a target for innovative therapeutic strategies. Future research is needed to further elucidate the precise neural mechanisms by which oxytocin exerts its effects, thereby paving the way for novel interventions that address social deficits and improve quality of life in affected individuals.

### The Default Mode Network: Structure and Function

The Default Mode Network (DMN) is a prominent large-scale brain network characterized by its heightened activity during rest and reduced activation during goal-directed tasks [4][26]. Its core regions include the medial prefrontal cortex (mPFC), posterior cingulate cortex (PCC), and angular gyrus, among others, which together support a range of internally directed cognitive processes such as autobiographical memory, self-referential thought, and future planning [27][28]. The discovery of the DMN has revolutionized our understanding of brain organization by highlighting that the brain is intrinsically active, even in the absence of overt external tasks [4][26][29].

Neuroimaging studies have consistently demonstrated that the DMN plays a critical role in integrating information related to the self, facilitating social cognition and introspection [30]. For instance, the mPFC is widely recognized for its involvement in processing self-relevant information and evaluating personal significance [31], while the PCC is implicated in the retrieval of autobiographical memories and integrating emotional information [32]. These regions collectively contribute to the brain’s capacity for "mental time travel," a process that allows individuals to project themselves into past experiences and future scenarios [33].

The DMN does not operate in isolation but rather interacts dynamically with other brain networks, such as the frontoparietal control network and the social brain network, to support complex cognitive functions [34]. Evidence suggests that these interactions facilitate flexible switching between internally and externally focused attention, which is critical for adaptive behavior [35]. Moreover, alterations in the connectivity patterns within the DMN have been linked to a range of neuropsychiatric conditions, underscoring the network’s importance in both normal and abnormal brain function [36][37].

Recent advances in functional connectivity analysis have allowed researchers to delineate the nuanced relationships between different DMN components, revealing both correlated and anticorrelated patterns of activity [38]. These findings have provided further insight into how the DMN contributes to semantic processing and the integration of sensory information with stored memories [39]. Overall, the functional architecture of the DMN underscores its vital role in supporting self-referential and social cognitive processes, offering a framework to understand how disruptions in this network may lead to clinical manifestations in various mental disorders [40].

### The Oxytocin-DMN Axis: Evidence and Mechanisms

Emerging evidence suggests that oxytocin modulates the connectivity within the Default Mode Network (DMN), thereby influencing social cognition and self-referential processing [41]. Intranasal oxytocin administration has been shown to alter the communication between core DMN nodes and limbic structures, such as the amygdala, which are essential for processing emotional salience [42]. The modulation of connectivity appears to be mediated by oxytocin receptor (OXTR) expression in key DMN regions, thereby facilitating synaptic plasticity and network integration [43]. Preclinical studies have demonstrated that oxytocin enhances synaptic plasticity in brain areas overlapping with the DMN, providing a mechanistic basis for its effects on network dynamics [44].

Human neuroimaging research has provided further insight into these modulatory effects. Functional magnetic resonance imaging (fMRI) studies reveal that oxytocin administration can increase functional connectivity between the medial prefrontal cortex and the posterior cingulate cortex, two critical nodes of the DMN [45]. These alterations in connectivity are thought to underpin enhanced integration of self-referential and social information processing [46]. In addition, advanced network analysis techniques have uncovered that oxytocin not only influences intra-DMN connectivity but also modulates the interaction between the DMN and other networks, such as the salience network and frontoparietal control network, which are vital for emotion regulation and executive function [47].

Clinical studies further underscore the relevance of the oxytocin-DMN axis. Disruptions in this axis have been implicated in neuropsychiatric conditions, including autism spectrum disorder and social anxiety disorder, where aberrant DMN connectivity correlates with the severity of social deficits [48]. Recent meta-analyses have supported the therapeutic potential of oxytocin as a neuromodulator agent capable of restoring typical DMN functionality in these clinical populations [49]. Future investigations employing multimodal imaging and pharmacological interventions are warranted to elucidate the precise molecular mechanisms by which oxytocin modulates DMN dynamics, and to further explore its therapeutic utility in treating mood and social disorders [50].

### Clinical Implications: Psychiatric Disorders and Dysregulation

Dysregulation of the oxytocin-DMN axis has been increasingly implicated in the pathophysiology of several psychiatric disorders marked by social dysfunction and impaired self-referential processing [51]. In autism spectrum disorder (ASD), for example, aberrant connectivity within the DMN combined with atypical oxytocin signaling may underlie deficits in social communication and empathy [52][53]. Similarly, individuals with social anxiety disorder often exhibit altered DMN patterns that correlate with heightened self-focused attention and negative self-evaluation, which may be further exacerbated by insufficient oxytocinergic modulation [54].

Major depressive disorder (MDD) has also been linked to disruptions in DMN connectivity, where excessive rumination and self-critical thoughts are thought to result from imbalanced network dynamics [55]. Emerging evidence suggests that oxytocin administration may help rebalance these network dynamics, potentially reducing depressive symptoms by enhancing social reward processing and modulating self-referential circuits [56]. In schizophrenia, the interplay between oxytocin and the DMN is of particular interest; preliminary studies indicate that oxytocin treatment may alleviate some of the social cognitive deficits associated with the disorder by normalizing DMN connectivity patterns [57][58].

Furthermore, the oxytocin-DMN relationship has been observed in studies examining parental behaviors, where disruptions in this axis are linked to impaired maternal responsiveness and bonding [59]. These findings suggest that therapeutic strategies targeting oxytocin signaling could offer promising avenues for ameliorating social and emotional dysfunctions across a range of conditions. Beyond clinical populations, insights from network neuroscience underscore that the integration and segregation of large-scale brain networks, including the DMN, are crucial for adaptive social behavior [60].

Overall, these clinical observations highlight the potential of modulating the oxytocin-DMN axis as a therapeutic intervention. Future research employing multimodal neuroimaging, genetic profiling, and controlled pharmacological studies is necessary to further elucidate the mechanistic underpinnings of this relationship and to optimize treatment strategies for disorders characterized by social and self-referential impairments.

### Therapeutic Potential and Future Directions

The emerging evidence on the modulation of the oxytocin-DMN axis has opened new avenues for therapeutic intervention in neuropsychiatric conditions marked by social deficits and impaired self-referential processing [61]. Clinical trials employing intranasal oxytocin have provided promising preliminary evidence that augmenting endogenous oxytocin levels can normalize aberrant DMN connectivity patterns and improve social cognition in disorders such as autism, social anxiety, and schizophrenia [62]. However, challenges remain regarding dosage optimization, administration protocols, and the identification of patient subgroups most likely to benefit from oxytocin-based therapies [63].

Preclinical models have been instrumental in elucidating the mechanistic underpinnings of oxytocin’s effects on brain networks. Rodent studies have demonstrated that oxytocin enhances synaptic plasticity and neurogenesis within DMN-associated regions, thereby facilitating improved cognitive and affective outcomes [64]. In parallel, advanced neuroimaging techniques have enabled the mapping of oxytocin-induced changes in functional connectivity across multiple neural circuits, suggesting that a network-level perspective may be critical in developing targeted interventions [65].

Future research is warranted to optimize the therapeutic potential of oxytocin, particularly by combining pharmacological treatments with behavioral interventions such as social skills training and cognitive-behavioral therapy [66]. Integrative approaches that harness the synergistic effects of neuroendocrine modulation and psychotherapy may offer the most effective strategies for restoring typical network dynamics and alleviating clinical symptoms [67].

Moreover, emerging technologies in brain stimulation, such as transcranial magnetic stimulation (TMS), present novel opportunities to modulate DMN activity directly, either alone or in combination with oxytocin administration [68]. Biomarker-driven studies using neuroimaging and genetic profiling will be crucial for personalizing treatment regimens and monitoring therapeutic efficacy [69].

Finally, longitudinal studies examining the long-term effects of oxytocin-based therapies on DMN connectivity and behavioral outcomes will be essential in validating these approaches and ensuring sustained benefits for patients [70]. As our understanding of the oxytocin-DMN axis deepens, it holds considerable promise for informing the development of innovative, individualized treatments that target the neural substrates of social cognition and emotional regulation.

## Conclusion

The exploration of the oxytocin-DMN axis has revealed a complex interplay between neuroendocrine modulation and intrinsic brain network dynamics that is critical for social cognition and self-referential processing. Evidence from preclinical studies, neuroimaging research, and clinical trials collectively underscores the potential of oxytocin to influence DMN connectivity and, consequently, behaviors associated with attachment, empathy, and emotional regulation. This body of work not only deepens our understanding of the neural substrates underlying social behavior but also opens promising avenues for the development of targeted therapeutic interventions for neuropsychiatric disorders characterized by social and self-referential impairments.

Future research should focus on refining oxytocin-based treatment protocols, integrating advanced neuroimaging techniques, and adopting personalized medicine approaches to better delineate the mechanisms underlying oxytocin’s effects on the DMN. Such efforts will be crucial in translating these findings into effective clinical applications that address the core deficits in social cognition and emotional processing, ultimately improving outcomes for individuals suffering from disorders like autism, social anxiety, depression, and schizophrenia.

### Conflicting Evidence and Moderating Factors

While numerous studies have demonstrated that oxytocin can enhance Default Mode Network (DMN) connectivity and promote prosocial behaviors, an emerging body of research suggests that its effects are not uniform. In some cases, the impact of oxytocin appears to be highly contingent on the social context in which it is administered. For instance, in supportive or affiliative settings, oxytocin may bolster trust and social bonding, whereas in contexts characterized by stress or perceived threat, it might instead heighten vigilance or even exacerbate negative social perceptions [71].

Individual differences further complicate this picture. Personality traits—such as baseline levels of social anxiety, attachment style, and even specific genetic polymorphisms related to oxytocin receptors—have been shown to modulate the neuropeptide’s effects on both behavior and neural connectivity. Such variability is particularly notable in clinical populations. In disorders like autism spectrum disorder, social anxiety, or depression, the neurobiological response to oxytocin can differ markedly, with some studies reporting beneficial outcomes while others find minimal or even paradoxical effects [72].

These conflicting findings underscore the complexity of the oxytocin-DMN axis and suggest that both environmental and individual factors play critical roles in determining the overall impact of oxytocin. This nuanced perspective calls for more personalized approaches in future research and clinical applications, ensuring that therapeutic interventions are tailored to the specific social and neurobiological profiles of individuals [71].

### Methodological Challenges and Future Directions

Despite promising findings, several methodological challenges constrain the interpretation of oxytocin studies. One major issue is the variability in intranasal administration techniques. Differences in dosage, delivery devices, and participant adherence can lead to inconsistent central nervous system uptake, making it difficult to compare results across studies.

Another challenge arises from the inherent variability in oxytocin receptor distribution among individuals. Genetic factors and receptor density differences may contribute to the heterogeneous effects observed, which complicates efforts to predict treatment outcomes or replicate findings reliably [73].

Moreover, current neuroimaging techniques used to assess DMN connectivity have their limitations. While fMRI provides valuable insights into functional connectivity, its temporal resolution and indirect measurement of neural activity may mask the dynamic and nuanced influence of oxytocin on brain networks. These technical constraints necessitate more advanced imaging modalities and multimodal approaches to fully capture the transient and context-dependent effects of oxytocin [74].

Addressing these methodological limitations will be critical for future research. Standardizing administration protocols, incorporating genetic screening, and utilizing complementary imaging techniques could help refine our understanding of the oxytocin-DMN axis and enhance the development of targeted therapeutic interventions [74].

### Translational Challenges and Future Therapeutic Directions

While the therapeutic potential of oxytocin to restore DMN functionality in psychiatric disorders is promising, significant challenges remain in translating these findings into clinical practice. Dosage optimization is a critical concern, as current research has yet to establish standardized protocols for achieving consistent central nervous system effects. Moreover, the long-term impact of repeated oxytocin administration remains largely unexplored, raising questions about its sustained efficacy and potential for unintended side effects [75]. These challenges underscore the need for comprehensive clinical trials that not only refine dosing strategies but also assess the safety profile over extended treatment periods. Future research may benefit from integrative approaches that combine oxytocin administration with behavioral therapies, such as cognitive-behavioral therapy or social skills training, to enhance therapeutic outcomes and provide more holistic interventions for disorders characterized by disrupted DMN connectivity [23].

### Interactions Beyond the DMN: Salience and Frontoparietal Control Networks

While the review primarily focuses on the Default Mode Network (DMN), it is important to recognize that oxytocin’s influence extends to other critical brain networks. The salience network, for example, plays a vital role in detecting and prioritizing behaviorally relevant stimuli, facilitating the switch between internally focused and externally directed attention. Similarly, the frontoparietal control network supports executive functions and adaptive decision-making [76]. Evidence suggests that oxytocin may modulate the connectivity not only within the DMN but also among these networks, promoting a coordinated interplay that underpins complex social cognition and emotional regulation. This integrative perspective implies that oxytocin could enhance the dynamic balance between self-referential processing (mediated by the DMN) and externally oriented cognitive control (facilitated by the salience and frontoparietal networks). Future research exploring these interactions will provide a more holistic understanding of how oxytocin orchestrates brain network dynamics to influence social behavior [76].

## Conflict of Interest

The author declares no conflict of interest.
